# Inbred Rats as a Model to Study Persistent Renal Leptospirosis and Associated Cellular Immune Responsiveness

**DOI:** 10.3389/fcimb.2018.00066

**Published:** 2018-03-14

**Authors:** Jarlath E. Nally, Jennifer H. Wilson-Welder, Richard L. Hornsby, Mitchell V. Palmer, David P. Alt

**Affiliations:** Infectious Bacterial Diseases Research Unit, National Animal Disease Center, Agricultural Research Service, United States Department of Agriculture, Ames, IA, United States

**Keywords:** *Leptospira*, spirochetes, persistent renal colonization, renal lymph node, CD4+ T cells

## Abstract

Pathogenic species of *Leptospira* cause leptospirosis, a bacterial zoonotic disease with a global distribution affecting over one million people annually. Rats are regarded as one of the most significant reservoir hosts of infection for human disease, and in the absence of clinical signs of infection, excrete large numbers of organisms in their urine. A unique biological equilibrium exists between pathogenic leptospires and reservoir hosts of infection, but surprisingly, little is known concerning the host's cellular immune response that facilitates persistent renal colonization. To address this deficiency, we established and applied an immunocompetent inbred rat model of persistent renal colonization; leptospires were detected in urine of experimentally infected rats by 3 weeks post-infection and remained positive until 8 weeks post-infection. However, there was little, if any, evidence of inflammation in colonized renal tubules. At 8 weeks post-infection, a robust antibody response was detected against lipopolysaccharide and protein outer membrane (OM) components. Purified B and T cells derived from the spleen of infected and non-infected rats proliferated in response to stimulation with 0.5 μg of OM fractions of *Leptospira*, including CD4+ T cells, which comprised 40% of proliferating cells, compared to 25% in non-infected controls. However, analysis of gene expression did not determine which immunoregulatory pathways were activated. Lymphocytes purified from the lymph node draining the site of colonization, the renal lymph node, also showed an increase in percentage of proliferating B and T cells. However, in contrast to a phenotype of 40% CD4+ T cells in the spleen, the phenotype of proliferating T cells in the renal lymph node comprised 65% CD4+ T cells. These results confirm that the renal lymph node, the local lymphoid organ, is a dominant site containing *Leptospira* reactive CD4+ T cells and highlight the need to consider the local, vs. systemic, immune responses during renal colonization infection. The use of inbred immunocompetent rats provides a novel tool to further elucidate those pathophysiological pathways that facilitate the unique biological equilibrium observed in reservoir hosts of leptospirosis.

## Introduction

Leptospirosis is a zoonotic disease of global significance caused by a unique group of bacteria (Bharti et al., 2003). Pathogenic species of *Leptospira* are excreted from colonized renal tubules of infected reservoir hosts via urine into the environment where they can survive in suitable moist conditions. Contact with urine from infected reservoir hosts, or contaminated water sources, can result in disease when pathogenic leptospires penetrate breaches of the skin, or mucosal surfaces, and disseminate haematogenously to cause a range of clinical symptoms from mild fever, to icteric Weil's disease and pulmonary hemorrhage syndrome. Mortality in these incidental hosts ranges from 10 to 70% (McBride et al., 2005). Leptospirosis is estimated to cause 1.03 million cases and 58,900 deaths each year (Torgerson et al., 2015). In developed countries, leptospirosis is primarily a recreational disease, an occupational disease of farm workers, veterinarians, and slaughter plant workers, or in returning travelers. In developing countries, it is a socioeconomic disease perpetuated by rapid urbanization, rodent infestation and transmission via contaminated water sources associated with limited infrastructures and severe weather events. Although rats are regarded as one of the most significant reservoir hosts of infection for human disease (Costa et al., 2014), many domestic animal species are also asymptomatic carriers, including dogs, cattle and pigs (Rojas et al., 2010; Ellis, 2015).

*Rattus norvegicus* was first recognized as a reservoir host of leptospirosis over 100 years ago, since experimental infection did not result in any clinical signs of disease, despite the persistent excretion of leptospires from kidney tissues which were subsequently lethal to guinea pigs (Ido et al., 1917). Though a unique biological equilibrium exists between pathogenic leptospires and reservoir hosts of infection, virtually nothing is known about those host-pathogen interactions that facilitate persistent renal colonization. There appears to be a specific host-parasite relationship in the Norway rat with serogroup Icterohaemorrhagiae, as compared to other serovars, since experimental infection results in persistent excretion over 220 days (Thiermann, 1981). Infected wild rats shed >10^6^ leptospires/ml of urine and their presence in households is significantly associated with the risk of infection (Costa et al., 2014, 2015).

Experimentally infected rats persistently excrete large numbers of leptospires in urine which has allowed for the characterization of urinary derived leptospires compared to its *in vitro* cultivated counterpart (Bonilla-Santiago and Nally, 2011). Leptospires excreted from renal tubules modify their protein and antigen expression, and regulate expression of protein post-translational modifications, a function hypothesized to help evade host immune responses (Nally et al., 2005, 2011, 2017; Monahan et al., 2008; Witchell et al., 2014). Antibodies from experimentally infected rats react with a larger number of antigens expressed by *in vitro* cultivated leptospires compared to urinary derived leptospires (Monahan et al., 2008). Urine from experimentally infected rats contains host-derived biomarkers of infection (Nally et al., 2015).

In the current study, we refined the rat model of persistent renal colonization to use immunocompetent inbred rats. Leptospires colonized renal tubules by 3 weeks post-infection and were persistently excreted at 8 weeks post-infection. Despite this, no pathology was observed in renal tissues. Experimentally infected rats produced antibody against protein and lipopolysaccharide antigens of leptospires. In addition, there was a cell specific proliferative immune response. Interestingly, lymphocytes derived from spleen responded differently to those derived from the renal lymph node, demonstrating the need to study not just systemic, but localized cellular immune responses. Our results emphasize the unique biological equilibrium observed between leptospires and their respective reservoir host of infection, and provide a framework to further understand the hosts' cellular immunoregulatory pathways associated with renal colonization.

## Materials and methods

### *In vitro* cultivated bacteria

Virulent low-passage *L. interrogans* serogroup Icterohaemorrhagiae strain RJ19115 was cultivated under standard conditions at 30°C in EMJH medium. Virulence was assessed by experimental infection of guinea pigs (Nally et al., 2004; Schuller et al., 2015).

### Experimental infection of rats and sample collection

All animal experimentation was conducted in accordance with protocols as reviewed and approved by the Animal Care & Use Committee at the National Animal Disease Center, and as approved by USDA Institutional guidelines. Fifteen female Fisher 344 inbred rats (Strain F344/NHsd, Envigo) of approximately 4 weeks of age were experimentally infected with 1 × 10^7^ low-passage *L. interrogans* strain RJ19115 by intraperitoneal injection in a final volume of 0.5 ml. From 3 to 6 weeks post-infection, urine was collected weekly from rats for enumeration of spirochetes by dark-field microscopy (DFM) as previously described (Miller, 1971). The limit of detection of leptospires by DFM is 10^5^ leptospires/ml. In order to collect urine samples, rats were housed individually in a metabolism cage immediately after receiving furosemide (2–10 mg/kg) intramuscularly (Bonilla-Santiago and Nally, 2011). Nine additional rats served as non-infected controls and received 0.5 ml of culture medium.

### Microscopic agglutination test

Serum was collected from each rat at approximately 8 weeks post-infection by cardiac puncture. The microscopic agglutination test (MAT) was performed using strain RJ19115, according to OIE guidelines at 2-fold dilutions from an initial dilution of 1:25 (Cole et al., 1973).

### Pathology and immunohistochemistry

Rat kidneys were harvested at approximately 8 weeks post-infection and immediately fixed by immersion in neutral buffered 10% formalin, processed routinely, embedded in paraffin, cut into 4 μm sections, and stained with hematoxylin and eosin (HE). Immunohistochemistry was performed on paraffin-embedded tissue sections using antiserum generated against outer membrane vesicles (OMV) of *Leptospira* species or with anti-LipL32 (Nally et al., 2004, 2017). After dewaxing, tissue sections were blocked with 10% normal goat serum in PBS for 30 mins at room temperature. Samples were incubated with anti-OMV or anti-LipL32 at 1:200 in blocking solution and incubated overnight at 4°C. After 3 × 5 min washes in PBS, samples were incubated in goat anti-rabbit IgG conjugated to AlexaFluor 546 (Invitrogen, CA) and DAPI (Invitrogen, CA) 1:3,000 in blocking solution for 60 min at room temperature in the dark. Samples were again washed in PBS before the addition of ProLong Gold anti-fade (Molecular Probes, OR) mounting media per slide and covered with a 24 × 50 mm coverslip. Samples were viewed using a Nikon Eclipse E800 and images captured using Nikon Elements Software.

### Antigen preparation and immunoblotting

Fractionation of *L. interrogans* strain RJ19115 to enrich for outer membrane (OM) proteins was performed using Triton X-114 as previously described (Nally et al., 2001). OM enriched fractions were compared to whole leptospires by 1-D gel electrophoresis as previously described (Monahan et al., 2008). Proteins were visualized by staining with Sypro Ruby (Invitrogen, CA) and lipopolysaccharide was visualized by staining with Pro-Q Emerald 300 (Invitrogen, CA) as per manufacturer's guidelines. For immunoblotting, samples were transferred to Immobilon-P transfer membrane (Millipore, 220 Bedford, MA) and blocked overnight at 4°C with StartingBlock (TBS) blocking buffer (Thermo Scientific, CO). Membranes were individually incubated with indicated antisera (anti-LipL21, anti-LipL32 and anti-LipL41 at 1:4,000, anti-*Treponema* FlaA at 1:2,000, or a pool of sera from infected or non-infected rats at 1:1,000, in PBS-T for 1 h at room temperature), followed by incubation with horseradish-peroxidase anti-rabbit immunoglobulin G conjugate or horseradish-peroxidase anti-rat immunoglobulin G conjugate (Sigma, MO). Bound conjugates were detected using Clarity Western ECL substrate (BioRad, CA) and images acquired using a Bio-Rad ChemiDoc MP imaging system.

### Lymphocyte isolation

Spleens and renal lymph nodes were harvested from rats at approximately 8 weeks post-infection and placed in transport media (DMEM [Gibco] supplemented with 5% fetal bovine serum [FBS], 2% Pen-Strep [10,000 U/mL, Gibco], 150 μg/ml gentamicin sulfate [Sigma]). Spleens were homogenized by pressing through a 40 μm mesh cell strainer with a syringe barrel plunger in a small volume of transport media. Homogenized spleen was overlaid onto Lympholyte®-Rat (Cedarlane, Canada). Renal lymph nodes were homogenized by gently mashing using the frosted end of two acid-washed autoclaved glass microscope slides, resuspended in PBS and overlaid onto Lympholyte®-Rat. Renal lymph nodes from 3 infected or non-infected animals were pooled together, while spleens were processed individually. After centrifugation, the lymphocyte cell layer was recovered and washed once with PBS. Red blood cells were then lysed by ammonium-chloride-potassium (ACK) lysis buffer (150 mM NH_4_Cl, 10 mM KHCO_3_, 0.01 mM Na_2_EDTA) for 90 s. Cells were passed through a 40 μm cell filter, washed by centrifugation, resuspended in PBS containing DNase (1 mg/mL, Sigma D25) for 10 min and washed again. Washed cells were resuspended in PBS and overlaid onto 5 ml FBS for a final wash. Cells were counted on a hemocytometer after staining with trypan blue. Cells were labeled with 10 nM Cell-Trace Violet (Molecular Probes) following manufacturer's recommendations and resuspended at a final concentration of 5 × 10^6^ per ml in DMEM media supplemented with 1% sodium pyruvate (Gibco), 2 mM L-glutamine (Gibco), 1% non-essential amino acids (Gibco), 10% FBS, 5 × 10^−3^ mM β-mercaptoethanol, 1% Pen-Strep (10,000 U/mL, Gibco) and 100 μg/ml gentamicin sulfate (Sigma). Cells were cultured in 96 well round bottom plates (5 × 10^5^ cells/well) in the presence of *L. interrogans* strain RJ19115 OM antigen at 0.5 μg/ml. This dose was experimentally determined to be optimal for stimulation. Concanavalin A (1 μg/ml) was included as a positive control stimulant, and media only was used as no stimulant/negative control. Cells were cultured for 4 days at 39°C with 5% CO_2_.

### Flow cytometry and statistical analysis

At 4 days post-stimulation in culture, cells were harvested by centrifugation, and labeled with live/dead discriminator dye (Zombie Yellow, Biolegend, CA) followed by antibodies to cell surface markers for CD3, CD4, CD8b, gamma-delta T cell receptor, NK T cell marker (CD161a), B220, and CD19. Primary antibodies, secondary antibodies, dilutions and suppliers are provided in Supplementary Table 1. Following labeling, cells were fixed (Stabilizing Fixative 3X, BD Bioscience, CA) and data collected using BD LSRII Flow Cytometer. Data analysis for cell phenotype was performed using FlowJo software with 2,000 cells within the live gate required for analysis. An example of gating strategy is provided in Supplementary Figure 1. Proliferation was indicated by a decrease in fluorescence intensity of cell membrane proliferation tracking dye as compared to no stimulation wells. Phenotype of proliferating subsets were determined with gate restrictions being set using fluorescence-minus-one. Data was further analyzed for statistical significance using GraphPad Prism 7 software fitting 2-way ANOVA with Sidak's multiple comparisons post-test, comparing within groups (Control or Infected) effect of well stimulation to no stimulation wells and between groups for a given well treatment. Mean percentages were significant if *p* ≤ 0.05. Statistical significance for cell phenotype between groups was determined by multiple *t*-tests using the Holm-Sidak method with alpha = 0.05.

### Gene expression arrays and statistical analysis

Spleens were harvested from rats at approximately 8 weeks post-infection and a portion flash frozen on dry ice. Samples were stored at −80°C until RNA extraction using the RNeasy RNA kit (Qiagen). Purified RNA was treated with Ambion Turbo Free DNase (Thermo-Fisher) and sample quality checked using the BioAnalyzer 2,100–RNA Chip Nano 6,000 (Agilent, CA). Two microgram of RNA was used per 150 μl cDNA synthesis reaction (Invitrogen Superscript IV First Strand Synthesis kit) for each animal. cDNA amounts were normalized and pooled such that 20 ng from three infected or non-infected animals provided 60 ng per each well of a BioRad Prime PCR custom array PCR assay plate. Genes assayed, relevant gene identification information, and primer sequence as supplied by the manufacturer are supplied in Supplementary Table 2. RT-PCR assays were performed using SSOAdvanced Universal SYBR Green Supermix (BioRad) on 384 CFX C1000 Touch Thermal Cycler (BioRad). QRT-PCR analysis was carried out using BioRad CFX Manager Software using Single Threshold and the relative gene expression levels were calculated using comparative Ct (ΔΔCt) method, normalized to the expression of 2 housekeeper genes (*actb, b2m*). Replicate wells with Ct standard deviations >0.5 were removed from further analysis. Gene expression was calculated as 2^−ΔΔCt^ and then Log_2_ transformed using GraphPad Prism 7 for statistical analysis. Statistical significance was determined by multiple *t*-tests using the Holm-Sidak method with alpha = 0.05. An expression ratio of 1.5 was chosen as difference from control (non-infected).

## Results

### Persistent excretion of leptospires

All experimentally infected rats were positive excretion of leptospires in urine, as detected by dark-field microscopy (DFM), by 3 weeks post-infection, Table 1. Numbers of leptospires in urine ranged from 1 × 10^5^ leptospires/ml (the lowest limit of detection by DFM) up to 1 × 10^7^ leptospires/ml. Kidneys from all experimentally infected rats were culture positive demonstrating persistent renal colonization until the end of the experiment. All infected rats displayed similar weight increases compared to non-infected controls (data not shown).

**Table 1:** Experimentally infected rats were positive for persistent renal leptospirosis.

**Animal #**	**Week 3**	**Week 4**	**Week 5**	**Week 6**	**Culture**	**MAT**
1	1.00E + 05	1.00E + 05	1.00E + 05	ND	+	1:800
2	5.00E + 06	1.00E + 07	5.00E + 06	3.00E + 06	+	1:1,600
3	1.00E + 06	1.00E + 06	3.00E + 06	3.00E + 06	+	1:1,600
4	5.00E + 06	8.00E + 06	3.00E + 06	4.00E + 06	+	1:1,600
5	3.00E + 06	2.00E + 06	2.00E + 06	3.00E + 06	+	1:800
6	1.00E + 05	1.00E + 05	1.00E + 05	ND	+	1:400
7	1.00E + 06	2.00E + 05	1.00E + 06	1.00E + 06	+	1:400
8	1.00E + 06	5.00E + 05	2.00E + 05	ND	+	1:800
9	3.00E + 06	3.00E + 06	1.00E + 06	4.00E + 06	+	1:800
10	5.00E + 06	2.00E + 06	1.00E + 06	1.00E + 06	+	1:1,600
11	7.00E + 05	1.00E + 06	1.00E + 06	1.00E + 06	+	1:400
12	1.00E + 07	5.00E + 06	2.00E + 06	1.00E + 06	+	1:1,600
13	1.00E + 06	2.00E + 05	2.00E + 06	3.00E + 06	+	1:800
14	1.00E + 05	2.00E + 05	1.00E + 05	ND	+	1:400
15	2.00E + 05	1.00E + 05	3.00E + 05	ND	+	1:400
Mean	2.56E + 06	2.36E + 06	1.53E + 06	2.18E + 06		

*Numbers of leptospires excreted per ml of urine, as detected by dark-field microscopy, at 3, 4, 5, and 6 weeks post-infection. At 8 weeks, all rats were kidney culture positive and MAT positive at indicated titer*.

*ND, Not detected*.

### Pathology and immunohistochemistry

**Figure 1:**
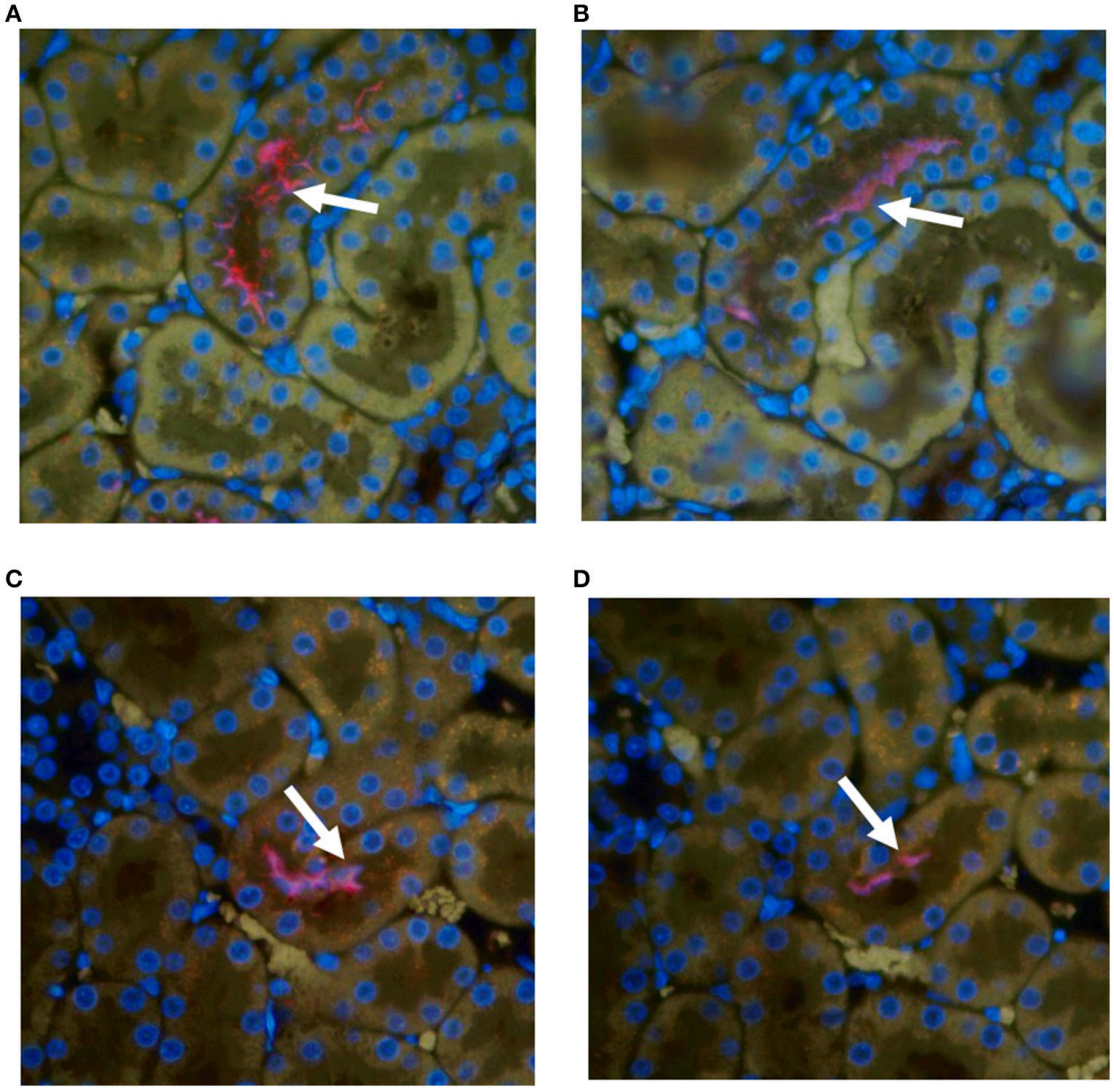
Representative immunohistochemical staining of kidneys from experimentally infected rats at 8 weeks post-infection with anti-OMV **(A,C)** or anti-LipL32 **(B,D)**. Arrows indicate positive renal tubules. Original magnification × 400.

All kidneys from experimentally infected rats were positive by immunohistochemistry using antibody specific for outer membrane vesicles (OMV) or anti-LipL32, Figure 1. In general, there was little, if any, evidence of inflammation. Direct comparison of areas positive for leptospires by immunofluorescence with their HE counterparts did not identify corresponding areas of inflammation. Occasionally, mild small foci of low numbers of interstitial lymphocytic infiltrates were observed in both infected and non-infected rats; none of these areas were positive for leptospires.

### Humoral response

**Figure 2:**
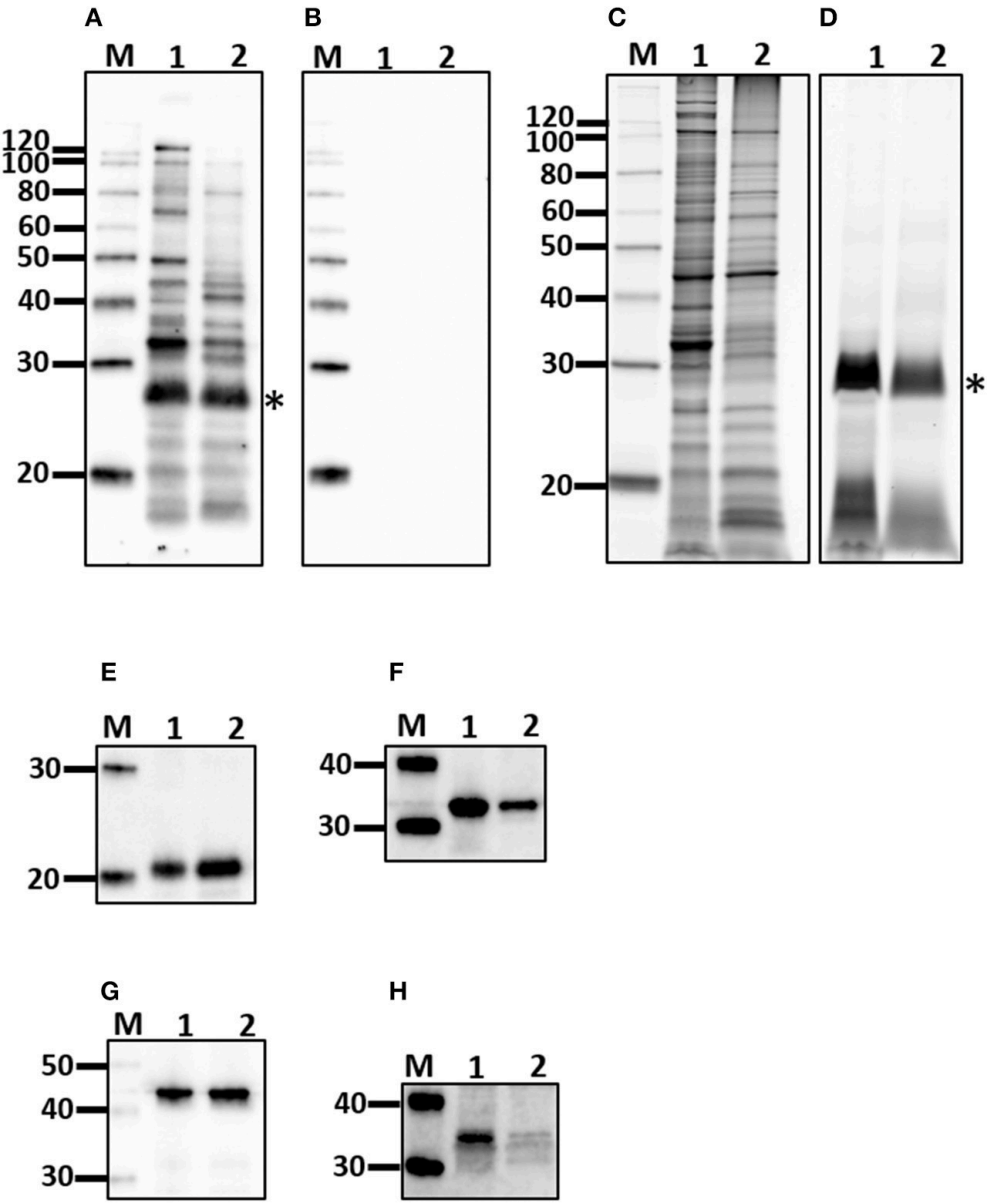
Characterization of outer membrane (OM) fraction. Antigen derived from whole cells (1) was compared to the OM fraction (2) by immunoblotting with sera from experimentally infected rats **(A)** or non-infected controls **(B)** and for protein **(C)**, and lipopolysaccharide content **(D)**, as well as the presence of LipL21 **(E)**, LipL32 **(F)**, LipL41 **(G)**, and FlaA **(H)**. ^*^Indicates O-antigen. Molecular mass markers (M) are indicated.

All experimentally infected rats had a positive MAT titer that ranged from 1:400 to 1:1,600 indicating a strong antibody response against leptospiral lipopolysaccharide, Table 1. A strong antibody response against leptospiral proteins was also evidenced by immunoblot, Figure 2A. Non-infected rats were negative by MAT (titer < 1:25) and immunoblot, Figure 2B.

### Cellular response

Purified lymphocytes were stimulated with a fraction of *Leptospira* enriched for outer membrane (OM) components. The interaction of OM proteins with the host during infection is indicated by a positive immunoblot, Figure 2A, compared to non-infected controls, Figure 2B. The protein content of the OM enriched fraction compared to the total protein profile of leptospires indicates those proteins that partitioned to the OM fraction, Figure 2C. The OM fraction contains lipopolysaccharide, Figure 2D, as well as a number of well characterized OM proteins including LipL21, LipL32, and LipL41, Figures 2E–G, respectively. As expected for the periplasmic FlaA, it was detected in minimal amounts in the OM fraction compared to whole leptospires, Figure 2H.

**Figure 3:**
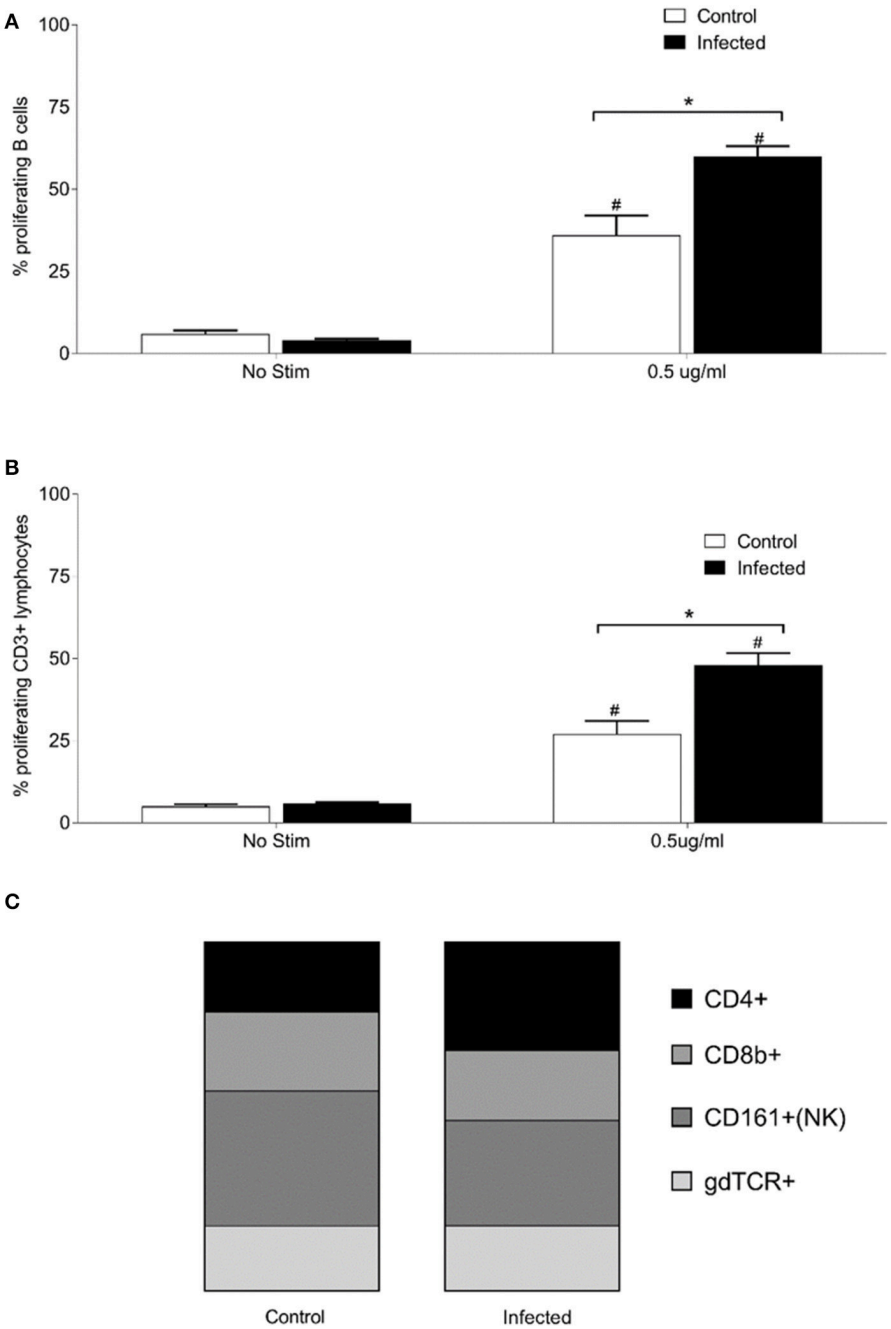
Proliferative responses and cellular phenotypes of lymphocytes isolated from the spleen of 15 infected and nine control rats. Percentage of proliferating B cells (CD3−, CD19+, or B220+) **(A)**, percentage of proliferating T cells (CD3+) **(B)** and phenotype by proportion of proliferating CD3+ cells stimulated with 0.5 μg/ml OM antigen **(C)**. ^*^indicates statistical significance between infected and control animals; ^#^Indicates statistical significance between OM antigen stimulated wells and no stimulant (No Stim) wells within the same group.

To examine relative numbers and phenotypes of antigen responsive cells, lymphocytes were isolated from chronically infected inbred rats. Purified lymphocytes derived from the spleen of 15 infected and nine non-infected rats proliferated in response to stimulation with 0.5 μg of OM fractions of *Leptospira*. Both infected and control animals showed a statistically significant (*p* < 0.001) increase in percentage of proliferating B cells (CD3−, B220+, or CD19+) and T cells (CD3+) when cultured in the presence of *Leptospira* OM fraction compared to lymphocytes cultured with media alone (No Stim), Figures 3A,B. Additionally, a significant (*p* < 0.001) increase was observed in the percentage of proliferating B-cells and T-cells isolated from spleens of infected rats when compared to non-infected controls. The phenotype of proliferating T cells stimulated with 0.5 μg OM fraction from infected animals comprised 40% CD4+ (standard deviation [SD] 8), 26% CD8b+ (SD 12), 39% NK/CD161a+ (SD 8), and 24% ⋎δ-TCR+ (SD 7), Figure 3C. The phenotype of proliferating T cells stimulated with 0.5 μg OM fraction from control animals comprised 25% CD4+ (SD 9), 28% CD8b+ (SD 12), 48% NK/CD161a+ (SD 12), and 23% ⋎δ-TCR+ (SD 7), Figure 3C. There was a statistical difference (*p* = 0.0013) in the percentage of proliferating CD4+ population between control and infected animals, indicating the presence and proliferation of *Leptospira* specific reactive CD4+ T cells in the spleen of persistently infected rats.

**Figure 4:**
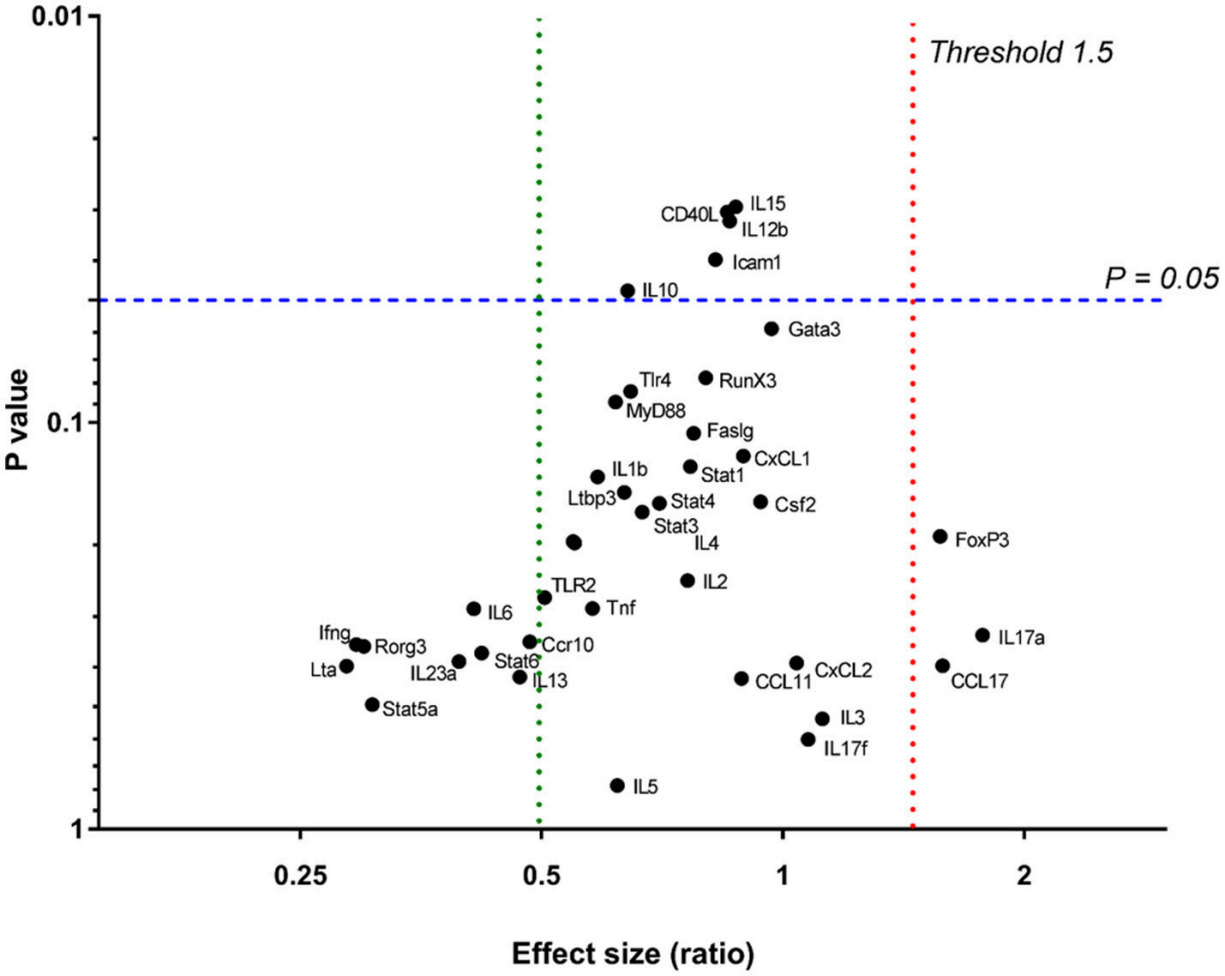
Relative gene expression in spleens of 15 infected vs. nine control rats. *P* = 0.05 is signified by a blue dashed line. The upper expression threshold (upregulated genes) (Effect size [ratio]) of 1.5 is signified by a red dotted line and lower expression (downregulated) threshold of 0.5, by a green dotted line.

In order to determine effector T cell functions or specific immune pathways that may have been induced by chronic *Leptospira* infection, gene expression for T-cell activation was analyzed in spleens of 15 infected and nine control rats. A custom T-cell activation panel was chosen to represent various T cell pathways including Th1, Th2, Th17, and Treg (Supplementary Table 2). As shown in Figure 4, none of the genes assayed were significantly differentially expressed (*p* < 0.05) above an expression threshold of 1.5.

**Figure 5:**
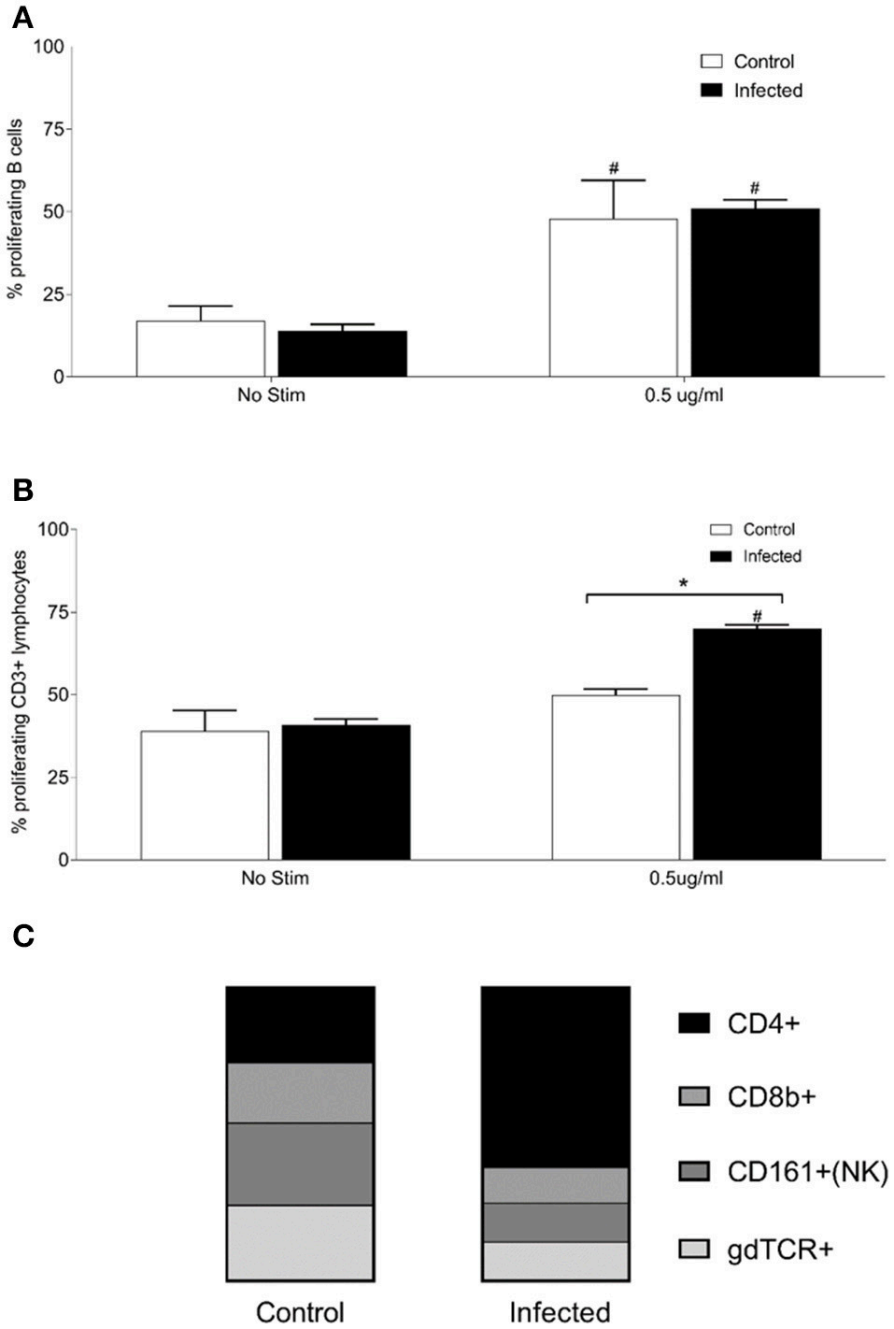
Proliferative responses and cellular phenotypes of lymphocytes isolated from the renal lymph node of 15 infected and nine control rats. Lymphocytes were isolated from the RLN, pooled into groups of 3 animals, and stimulated with purified OM fraction from *Leptospira*. Percentage of proliferating B cells (CD3−, CD19+, or B220+) **(A)**, percentage of proliferating T cells (CD3+) **(B)** and phenotype by proportion of proliferating CD3+ cells stimulated with 0.5 μg/ml OM antigen **(C)**. ^*^Indicates statistical significance between infected and control; ^#^Indicates statistical significance between OM antigen stimulated wells and no stimulant (No Stim) wells within the same group.

Given that the host response may be more localized to the site of colonization by *Leptospira*, antigen responsive lymphocytes derived from the renal lymph node (RLN) of 15 experimentally infected rats, and nine non-infected controls, were assayed. Lymphocytes were isolated from the RLN, pooled into groups of three animals, and stimulated with purified OM fraction from *Leptospira*. Both infected and control rats showed a significant increase (*p* < 0.005) in percentage of proliferating B cells (CD3−, CD19+, or B220+) when stimulated with 0.5 μg/ml OM antigen compared to no stimulant controls, Figure 5A, but no difference was observed between infected and control animals. Lymphocytes from infected RLN showed a significant increase in proliferating T cells (CD3+) compared to control RLN (*p* = 0.007) and no stimulant controls (*p* = 0.0069; Figure 5B). The phenotype of proliferating T cells stimulated with 0.5 μg OM fraction in RLN from infected animals comprised 65% CD4+ (SD 1), 13% CD8b+ (SD 3), 14% NK/CD161a+ (SD 5), and 14% ⋎δ-TCR+ (SD 1), Figure 5C. The phenotype of proliferating T cells stimulated with 0.5 μg OM fraction in RLN from control animals comprised 30% CD4+ (SD, 4), 24% CD8b+ (SD 3), 33% NK/CD161a+ (SD 1), and 30% ⋎δ-TCR+ (SD 1), Figure 5C. There was a statistical difference in all the T cell subsets between control and infected animals (*p* < 0.001), with the greatest difference being between the proportion of CD4+ subsets. These results confirm that the RLN, the local lymphoid organ, is the site containing the majority of the *Leptospira* reactive CD4+ T cells.

## Discussion

Host-pathogen relationships have evolved over millennia and range from asymptomatic, chronic and persistent carriage in some hosts compared to acute, fulminant disease in others. The unique biological equilibrium that exists between reservoir hosts of infection and bacterial pathogens is facilitated in part by the ability of the pathogen to express appropriate virulence factors that maintain infection, yet minimize detection and subsequent clearance by host immune responses. *Rattus norvegicus* is one of the most important reservoir hosts for the persistent dissemination and transmission of pathogenic leptospires to human populations throughout the world. Wild rats can routinely excrete >10^6^ leptospires/ml of urine, yet they also show evidence of an antibody response against lipopolysaccharide, and a cellular immune response as evidenced by interstitial nephritis (Tucunduva de Faria et al., 2007; Costa et al., 2015). In this manuscript, an immunocompetent inbred rat model of leptospirosis was developed to specifically address the host cellular immune responses associated with persistent renal infection.

Acute leptospirosis in humans and domestic animals is generally described as a biphasic disease (Haake and Levett, 2015). Phase one comprises a leptospiraemia during which leptospires disseminate throughout the host and thus cause a wide range of clinical symptoms, ranging from fever to pulmonary hemorrhage. The second convalescent phase is associated with the appearance of agglutinating antibodies which are believed to mediate clearance of leptospires. At this time, leptospires are no longer detected in blood but may be detected in urine. Urinary excretion in incidental hosts tends to be short-lived. A similar profile is observed in laboratory animal models of acute leptospirosis, including hamsters and guinea pigs, whereby an acute lethal disease is associated with dissemination of leptospires throughout the host; this is accompanied by significant weight loss and pathology in a range of tissues which can include lung, liver and kidney (Nally et al., 2004; Coutinho et al., 2011; Wunder et al., 2016). Experimental infection of *Rattus norvegicus* is also associated with an initial leptospiraemic phase during which leptospires are detected in liver, kidney and skeletal muscle but, in contrast to acute infection, no underlying histopathology, or weight loss, is observed (Athanazio et al., 2008; Monahan et al., 2008). By day 9 post-infection, leptospires are cleared from all organs except the kidney, and experimentally infected rats can become persistent urinary shedders of leptospires for months (Thiermann, 1981; Rojas et al., 2010; Bonilla-Santiago and Nally, 2011). Similarly, we now report that inbred immunocompetent *Rattus norvegicus* (Fisher 344) are susceptible to infection with *L. interrogans* serogroup Icterohaemorrhagiae as characterized by persistent renal colonization and urinary shedding for at least 8 weeks post-infection in the absence of pathology or weight loss.

Urinary excretion of leptospires at 8 weeks post-infection was evident despite the detection of a robust antibody response specific for both lipopolysaccharide and protein antigens (Table 1 and Figure 2). Antibody specific for the O-antigen of leptospiral polysaccharide is considered protective (Challa et al., 2011). The production of antibody was supported by the detection of proliferating B cells in the spleen of infected rats when stimulated with OM antigen. Experimental models using B cell deficient mice, suffering acute infection, have shown that B cells are a crucial lymphocyte subset responsible for the clearance of leptospires from liver and peripheral tissues (Chassin et al., 2009). Polymeric bacterial antigens, such as LPS, can bind directly to B cell receptors and induce B cell activation, even in the absence of the cognate T cell (Murphy and Weaver, 2016). *Leptospira* specific antibody may be found in the urine (Nally et al., 2011), however, the lack of innate or other cell-types, which interact with pathogen bound antibody for an effector function, within the undamaged renal tubule makes the antibody of little consequence to the pathogen (Lu et al., 2018). Antigen-specific proliferating CD3+ T cells were evidenced in spleen and included CD4+, CD8+, NK, and ⋎δ-TCR+. The proportion of CD4+ CD3+ antigen stimulated proliferating cells increased from 25 to 40% in those spleens derived from infected rats compared to non-infected controls (Figure 3). In order to identify those immunoregulatory pathways involved in cellular activation, gene expression profiles of spleen tissue from infected or non-infected control rats were assayed with a custom gene expression array that included genes previously shown to regulate T cell pathways, including Th1, Th2, Treg, and Th17 (Supplementary Table 2; Belkaid and Rouse, 2005; Iwakura et al., 2008; Gasteiger and Rudensky, 2014; Godfrey et al., 2015). No differences in gene expression values were observed in the spleen from infected and control animals (Figure 4). This may be a result of multiple factors including (1) the lack of pathology observed in the kidney despite the presence of leptospires which suggests a limited activation of the host response, (2) the limited number of genes assayed (3) the low numbers of T cells induced in the spleen in this model of infection, or (4) the selected time point post-infection for analysis. These observations are in keeping with an increase in levels of gene expression for both IL17a, an indicator of pro-inflammatory immune responses and FoxP3, a transcription factor associated with the development of T-regulatory cells, in infected rat spleens that failed to reach statistical levels of significance (Figure 4). Analysis of differential gene expression at earlier time points post-infection may provide additional insights.

Given that the presence of leptospires in the rat was limited to renal tissues, lymphocytes were also purified from the lymph node draining the site of colonization, the renal lymph nodes. As with the spleen, proliferating B cells were readily detected in stimulated cells, though similar profiles were observed whether they were derived from infected or non-infected animals. However, there was also a readily detected antigen specific increase in proliferating CD3+ T-cells from the renal lymph node of infected rats compared to non-infected rats (Figure 5); the percentage of antigen proliferating CD4+ T cells increased from 30 to 65% compared to other cell types; CD8+ T cells decreased from 24 to 13%, NK cells from 33 to 14% and ⋎δ-TCR+ from 30 to 14%. Additional phenotypic markers and/or cytokine analysis will be required to determine whether the memory phenotype or functionality (Th1-Th2-Th17) of these antigen-reactive CD4+ T cells in the spleen are similar to those in the renal lymph nodes of chronically infected rats.

A predominant lesion in naturally infected chronic rat leptospirosis is diffuse interstitial nephritis (Sterling and Thiermann, 1981; Tucunduva de Faria et al., 2007; Monahan et al., 2009; Agudelo-Flórez et al., 2013). In rats experimentally infected with 10^8^ *L. interrogans* serovar Copenhageni, interstitial nephritis was detected in 33% of rats by 4 weeks post-infection (Tucunduva de Faria et al., 2007); by 2 months, this number increased to 71.4% thus highlighting the dynamic nature of the cellular immune response. No interstitial nephritis was observed during our study at 8 weeks-post infection though this may be due to the lower dose of 10^7^ leptospires used in our study. However, interstitial nephritis has been observed at 160 days post-infection in rats experimentally infected with a lower dose of 10^6^ leptospires. At this time, a lymphocyte-rich inflammatory infiltrate in association with leptospires in infected kidney sections was apparent; however, it was also apparent that leptospiral organisms were also detected within tubules devoid of any immune response. It remains unclear if such responses can ultimately be successful enough to remove organisms and eliminate renal excretion, or whether leptospires can continue to evade detection and reactivity as hypothesized by differential expression of antigens (Nally et al., 2007, 2011, 2017; Monahan et al., 2008; Witchell et al., 2014).

At 8 weeks post-infection, all rats had a positive MAT titre ranging from 1:400 to 1:1600. This differs to experimentally infected rats that were MAT negative at 160 days post-infection (Nally et al., 2015). This discordance is likely due to waning titers over time since experimentally infected rats were exposed to infection only once, compared to wild type rats, which appear to maintain titers, and are routinely exposed via multiple routes (Costa et al., 2015; Minter et al., 2017). The MAT is typically used to diagnose acute disease; in the absence of clinical signs of infection, it only serves to indicate exposure, and is of limited use to detect domestic animals that are acting as reservoir hosts of infection. Cattle, acting as reservoir hosts for serovar Hardjo, are routinely MAT negative (Miller et al., 1991; Libonati et al., 2017). The leading cause of bovine leptospirosis is infection with serovar Hardjo, including both members of *L. interrogans* and *L. borgpetersenii*. Most bovine infections are inapparent clinically, despite the potential shedding of viable *Leptospira* for extended periods of time. Isolation, although successful from bovine reproductive tissues, is most frequently accomplished as seen here with the rat, from either the kidney or from urine. Although interstitial nephritis can be detected in the kidneys of cattle experimentally infected with *L. borgpetersenii* serovar Hardjo, lesions produced are usually minimal in scope and limited to focal capsular depressions or areas of pallor. Histologically, these foci may show evidence of tubular and glomerular damage with inflammatory infiltrates and fibrosis (Bolin and Alt, 2001; Zuerner et al., 2011). Protective vaccine-induced responses in cattle tend to favor a Th-1 immune response involving both CD4+ T cells and ⋎δ-T cells (Naiman et al., 2002).

Studies on the cell-mediated immune response to leptospirosis have been limited to humans and small animal models with acute symptoms. Hamsters and humans, which exhibit acute disease, express high levels of pro-inflammatory cytokines IL-6 and TNFα in infected tissues or from PBMC (Matsui et al., 2011; Volz et al., 2015). Increased levels of IL-10, while also highly expressed in infected hamsters, were associated with milder disease in humans (Raffray et al., 2015). Humans with higher bacterial burdens had decreased numbers of circulating ⋎δ-T cells (Raffray et al., 2015). However, a C3H/HeJ mouse model of nonlethal acute disease, induced a more Th2-mediated CD4+ effector response (Richer et al., 2015). Much of the discrepancy in conflicting reports comes from trying to draw parallels between not just different species, but different types of infection: mild to sublethal to acute lethal disease. The rat, an asymptomatic reservoir host, does produce antibody and a robust cellular response at the draining lymph node, despite an apparent lack of inflammatory responses. While a Th1 vs. a Th2 type response was not elucidated in this current study, it may be that chronic leptospirosis in the reservoir host is similar to that of syphilis and starts out as a Th1 response with robust antibody; with time, and as the disease changes tissue tropism, the immune response shifts to a more Th2-like response as the pathogen and host reach biological equilibrium (Fitzgerald, 1992).

Regardless, the use of an inbred immunocompetent rat model of persistent renal colonization, using different doses of inoculum at a range of time-points post-infection, provides for the further characterization of the host cellular immune response, in both systemic and local immune sites, to understand the pathophysiology of persistent renal colonization over time in an immunocompetent reservoir host of infection.

## Author contributions

Conceived and designed the experiments: JN and JW-W. Performed the experiments: JN, JW-W, and RH. Contributed resources, reagents, materials, and analysis tools: JN, JW-W, RH, MP, and DA. Wrote the paper: JN. Revised the paper: JN, JW-W, RH, MP, and DA.

### Conflict of interest statement

The authors declare that the research was conducted in the absence of any commercial or financial relationships that could be construed as a potential conflict of interest.

## Supplementary material

Supplementary Figure 1 Gating strategy for flow cytometry. A representative example using lymphocytes purified from the spleen of experimentally infected rats is provided. Live cells were gated using Zombie Yellow Live/Dead discriminator dye **(A)**. Live cells were then gated on forward scatter (FSC) and side scatter (SSC) pattern consistent with proliferating lymphocytes **(B)**. Proliferating lymphocytes were gated on decrease in fluorescence of Cell Trace Violet cellular proliferation dye **(C)**. Dotted line is unstimulated cells, gray histogram is ConA 1 μg/ml stimulated cells and black is 0.5 μg/ml OM antigen stimulated cells. Proliferating cells were then gated into CD3+ and CD3– populations against an irrelevant marker **(D)**. Proliferating B cell population was defined in CD3– subset as being CD19+ and/or CD45R/B220+ **(E)**. Proliferating T cell subset (CD3+) was further phenotyped into CD4+ vs. CD8b+ **(F)**, gamma-delta TCR+ (⋎δTCR) **(G)** and NK (CD161a+) **(H)** subsets respectively.

Supplementary Table 1 Antibodies used for flow cytometry.

Supplementary Table 2 List of genes used in custom RT-PCR array.

## References

[B1] Agudelo-Flórez P. Murillo V. E. Londoño A. F. Rodas J. D. (2013). Histopathological kidney alterations in rats naturally infected with *Leptospira*. Biomedica 33(Suppl. 1), 82–88. 10.7705/biomedica.v33i0.686 24652252

[B2] Athanazio D. A. Silva E. F. Santos C. S. Rocha G. M. Vannier-Santos M. A. McBride A. J.. (2008). *Rattus norvegicus* as a model for persistent renal colonization by pathogenic *Leptospira interrogans*. Acta Trop. 105, 176–180. 10.1016/j.actatropica.2007.10.012 18093568

[B3] Belkaid Y. Rouse B. T. (2005). Natural regulatory T cells in infectious disease. Nat. Immunol. 6, 353–360. 10.1038/ni1181 15785761

[B4] Bharti A. R. Nally J. E. Ricaldi J. N. Matthias M. A. Diaz M. M. Lovett M. A.. (2003). Leptospirosis: a zoonotic disease of global importance. Lancet Infect. Dis. 3, 757–771. 10.1016/S1473-3099(03)00830-2 14652202

[B5] Bolin C. A. Alt D. P. (2001). Use of a monovalent leptospiral vaccine to prevent renal colonization and urinary shedding in cattle exposed to *Leptospira borgpetersenii* serovar hardjo. Am. J. Vet. Res. 62, 995–1000. 10.2460/ajvr.2001.62.995 11453500

[B6] Bonilla-Santiago R. Nally J. E. (2011). Rat model of chronic leptospirosis. Curr. Protoc. Microbiol. Chapter 12:Unit12E.3. 10.1002/9780471729259.mc12e03s20 21400676

[B7] Challa S. Nally J. E. Jones C. Sheoran A. S. (2011). Passive immunization with *Leptospira* LPS-specific agglutinating but not non-agglutinating monoclonal antibodies protect guinea pigs from fatal pulmonary hemorrhages induced by serovar Copenhageni challenge. Vaccine 29, 4431–4434. 10.1016/j.vaccine.2011.04.041 21549788

[B8] Chassin C. Picardeau M. Goujon J. M. Bourhy P. Quellard N. Darche S.. (2009). TLR4- and TLR2-mediated B cell responses control the clearance of the bacterial pathogen, *Leptospira interrogans*. J. Immunol. 183, 2669–2677. 10.4049/jimmunol.0900506 19635914

[B9] Cole J. R. Sulzer C. R. Pursell A. R. (1973). Improved microtechnique for the leptospiral microscopic agglutination test. Appl. Microbiol. 25, 976–980. 4736794 10.1128/am.25.6.976-980.1973 PMC380950

[B10] Costa F. Ribeiro G. S. Felzemburgh R. D. Santos N. Reis R. B. Santos A. C.. (2014). Influence of household rat infestation on *Leptospira* transmission in the urban slum environment. PLoS Negl. Trop. Dis. 8:e3338. 10.1371/journal.pntd.0003338 25474580 PMC4256176

[B11] Costa F. Wunder E. A. Jr. De Oliveira D. Bisht V. Rodrigues G. Reis M. G.. (2015). Patterns in leptospira shedding in norway rats (*Rattus norvegicus*) from Brazilian Slum Communities at high risk of disease transmission. PLoS Negl. Trop. Dis. 9:e0003819. 10.1371/journal.pntd.0003819 26047009 PMC4457861

[B12] Coutinho M. L. Choy H. A. Kelley M. M. Matsunaga J. Babbitt J. T. Lewis M. S.. (2011). A LigA three-domain region protects hamsters from lethal infection by *Leptospira interrogans*. PLoS Negl. Trop. Dis. 5:e1422. 10.1371/journal.pntd.0001422 22180800 PMC3236721

[B13] Ellis W. A. (2015). Animal leptospirosis. Curr. Top. Microbiol. Immunol. 387, 99–137. 10.1007/978-3-662-45059-8_6 25388134

[B14] Fitzgerald T. J. (1992). The Th1/Th2-like switch in syphilitic infection: is it detrimental? Infect. Immun. 60, 3475–3479. 1386838 10.1128/iai.60.9.3475-3479.1992 PMC257347

[B15] Gasteiger G. Rudensky A. Y. (2014). Interactions between innate and adaptive lymphocytes. Nat. Rev. Immunol. 14, 631–639. 10.1038/nri3726 25132095 PMC4504695

[B16] Godfrey D. I. Uldrich A. P. McCluskey J. Rossjohn J. Moody D. B. (2015). The burgeoning family of unconventional T cells. Nat. Immunol. 16, 1114–1123. 10.1038/ni.3298 26482978

[B17] Haake D. A. Levett P. N. (2015). Leptospirosis in humans. Curr. Top. Microbiol. Immunol. 387, 65–97. 10.1007/978-3-662-45059-8_5 25388133 PMC4442676

[B18] Ido Y. Hoki R. Ito H. Wani H. (1917). The rat as a carrier of *Spirocheta icterohaemorrhagiae*, the causative agent of Weil's disease (spirochaetosis icterohaemorrhagica. J. Exp. Med. 26, 341–353. 10.1084/jem.26.3.341 19868153 PMC2125787

[B19] Iwakura Y. Nakae S. Saijo S. Ishigame H. (2008). The roles of IL-17A in inflammatory immune responses and host defense against pathogens. Immunol. Rev. 226:57–79. 10.1111/j.1600-065X.2008.00699.x 19161416

[B20] Libonati H. Pinto P. S. Lilenbaum W. (2017). Seronegativity of bovines face to their own recovered leptospiral isolates. Microb. Pathog. 108, 101–103. 10.1016/j.micpath.2017.05.001 28478204

[B21] Lu L. L. Suscovich T. J. Fortune S. M. Alter G. (2018). Beyond binding: antibody effector functions in infectious diseases. Nat. Rev. Immunol. 18, 46–61. 10.1038/nri.2017.106 29063907 PMC6369690

[B22] Matsui M. Rouleau V. Bruyère-Ostells L. Goarant C. (2011). Gene expression profiles of immune mediators and histopathological findings in animal models of leptospirosis: comparison between susceptible hamsters and resistant mice. Infect. Immun. 79, 4480–4492. 10.1128/IAI.05727-11 21844232 PMC3257942

[B23] McBride A. J. Athanazio D. A. Reis M. G. Ko A. I. (2005). Leptospirosis. Curr. Opin. Infect. Dis. 18, 376–386. 10.1097/01.qco.0000178824.05715.2c 16148523

[B24] Miller D. A. Wilson M. A. Beran G. W. (1991). Survey to estimate prevalence of *Leptospira interrogans* infection in mature cattle in the United States. Am. J. Vet. Res. 52, 1761–1765. 1785719

[B25] Miller J. N. (1971). Spirochetes in Body Fluids and Tissues. Springfield, IL: Charles, C. THomas.

[B26] Minter A. Diggle P. J. Costa F. Childs J. Ko A. I. Begon M. (2017). Evidence of multiple intraspecific transmission routes for *Leptospira* acquisition in Norway rats (*Rattus norvegicus*). Epidemiol. Infect. 145, 3438–3448. 10.1017/S0950268817002539 29173242 PMC6252042

[B27] Monahan A. M. Callanan J. J. Nally J. E. (2008). Proteomic analysis of *Leptospira interrogans* shed in urine of chronically infected hosts. Infect. Immun. 76, 4952–4958. 10.1128/IAI.00511-08 18765721 PMC2573331

[B28] Monahan A. M. Callanan J. J. Nally J. E. (2009). Review paper: Host-pathogen interactions in the kidney during chronic leptospirosis. Vet. Pathol. 46, 792–799. 10.1354/vp.08-VP-0265-N-REV 19429975

[B29] Murphy K. Weaver C. (2016). Janeway's Immunobiology. New York, NY: Garland Science.

[B30] Naiman B. M. Blumerman S. Alt D. Bolin C. A. Brown R. Zuerner R.. (2002). Evaluation of type 1 immune response in naïve and vaccinated animals following challenge with *Leptospira borgpetersenii* serovar Hardjo: involvement of WC1+ γδ and CD4 T cells. Infect. Immun. 70, 6147–6157. 10.1128/IAI.70.11.6147-6157.2002 12379692 PMC130359

[B31] Nally J. E. Artiushin S. Timoney J. F. (2001). Molecular characterization of thermoinduced immunogenic proteins Qlp42 and Hsp15 of *Leptospira interrogans*. Infect. Immun. 69, 7616–7624. 10.1128/IAI.69.12.7616-7624.2001 11705941 PMC98855

[B32] Nally J. E. Chantranuwat C. Wu X. Y. Fishbein M. C. Pereira M. M. Da Silva J. J.. (2004). Alveolar septal deposition of immunoglobulin and complement parallels pulmonary hemorrhage in a guinea pig model of severe pulmonary leptospirosis. Am. J. Pathol. 164, 1115–1127. 10.1016/S0002-9440(10)63198-7 14982864 PMC1614724

[B33] Nally J. E. Chow E. Fishbein M. C. Blanco D. R. Lovett M. A. (2005). Changes in lipopolysaccharide O antigen distinguish acute versus chronic *Leptospira interrogans* infections. Infect. Immun. 73, 3251–3260. 10.1128/IAI.73.6.3251-3260.2005 15908349 PMC1111870

[B34] Nally J. E. Grassmann A. A. Planchon S. Sergeant K. Renaut J. Seshu J.. (2017). Pathogenic leptospires modulate protein expression and post-translational modifications in response to mammalian host signals. Front. Cell. Infect. Microbiol. 7:362. 10.3389/fcimb.2017.00362 28848720 PMC5553009

[B35] Nally J. E. Monahan A. M. Miller I. S. Bonilla-Santiago R. Souda P. Whitelegge J. P. (2011). Comparative proteomic analysis of differentially expressed proteins in the urine of reservoir hosts of leptospirosis. PLoS ONE 6:e26046. 10.1371/journal.pone.0026046 22043303 PMC3197145

[B36] Nally J. E. Mullen W. Callanan J. J. Mischak H. Albalat A. (2015). Detection of urinary biomarkers in reservoir hosts of leptospirosis by capillary electrophoresis-mass spectrometry. Proteomics Clin. Appl. 9, 543–551. 10.1002/prca.201400205 25736478

[B37] Nally J. E. Whitelegge J. P. Bassilian S. Blanco D. R. Lovett M. A. (2007). Characterization of the outer membrane proteome of *Leptospira interrogans* expressed during acute lethal infection. Infect. Immun. 75, 766–773. 10.1128/IAI.00741-06 17101664 PMC1828474

[B38] Raffray L. Giry C. Thirapathi Y. Binois F. Moiton M. P. Lagrange-Xelot M.. (2015). High leptospiremia is associated with low gamma-delta T cell counts. Microbes Infect. 17, 451–455. 10.1016/j.micinf.2015.04.001 25899947

[B39] Richer L. Potula H. H. Melo R. Vieira A. Gomes-Solecki M. (2015). Mouse model for sublethal *Leptospira interrogans* infection. Infect. Immun. 83, 4693–4700. 10.1128/IAI.01115-15 26416909 PMC4645400

[B40] Rojas P. Monahan A. M. Schuller S. Miller I. S. Markey B. K. Nally J. E. (2010). Detection and quantification of leptospires in urine of dogs: a maintenance host for the zoonotic disease leptospirosis. Eur. J. Clin. Microbiol. Infect. Dis. 29, 1305–1309. 10.1007/s10096-010-0991-2 20559675

[B41] Schuller S. Sergeant K. Renaut J. Callanan J. J. Scaife C. Nally J. E. (2015). Comparative proteomic analysis of lung tissue from guinea pigs with Leptospiral Pulmonary Haemorrhage Syndrome (LPHS) reveals a decrease in abundance of host proteins involved in cytoskeletal and cellular organization. J. Proteomics 122, 55–72. 10.1016/j.jprot.2015.03.021 25818725

[B42] Sterling C. R. Thiermann A. B. (1981). Urban rats as chronic carriers of leptospirosis: an ultrastructural investigation. Vet. Pathol. 18, 628–637. 10.1177/030098588101800508 7281461

[B43] Thiermann A. B. (1981). The Norway rat as a selective chronic carrier of *Leptospira icterohaemorrhagiae*. J. Wildl. Dis. 17, 39–43. 10.7589/0090-3558-17.1.39 7253100

[B44] Torgerson P. R. Hagan J. E. Costa F. Calcagno J. Kane M. Martinez-Silveira M. S.. (2015). Global burden of leptospirosis: estimated in terms of disability adjusted life years. PLoS Negl. Trop. Dis. 9:e0004122. 10.1371/journal.pntd.0004122 26431366 PMC4591975

[B45] Tucunduva de Faria M. Athanazio D. A. Gonçalves Ramos E. A. Silva E. F. Reis M. G. Ko A. I. (2007). Morphological alterations in the kidney of rats with natural and experimental Leptospira infection. J. Comp. Pathol. 137, 231–238. 10.1016/j.jcpa.2007.08.001 17996544

[B46] Volz M. S. Moos V. Allers K. Luge E. Mayer-Scholl A. Nöckler K.. (2015). Specific CD4+ T-Cell reactivity and cytokine release in different clinical presentations of leptospirosis. Clin. Vaccine Immunol. 22, 1276–1284. 10.1128/CVI.00397-15 26491036 PMC4658588

[B47] Witchell T. D. Eshghi A. Nally J. E. Hof R. Boulanger M. J. Wunder E. A. Jr.. (2014). Post-translational modification of LipL32 during *Leptospira interrogans* infection. PLoS Negl. Trop. Dis. 8:e3280. 10.1371/journal.pntd.0003280 25356675 PMC4214626

[B48] Wunder E. A. Jr. Figueira C. P. Santos G. R. Lourdault K. Matthias M. A. Vinetz J. M.. (2016). Real-Time PCR reveals rapid dissemination of *Leptospira interrogans* after intraperitoneal and conjunctival inoculation of hamsters. Infect. Immun. 84, 2105–2115. 10.1128/IAI.00094-16 27141082 PMC4936353

[B49] Zuerner R. L. Alt D. P. Palmer M. V. Thacker T. C. Olsen S. C. (2011). A *Leptospira borgpetersenii* serovar Hardjo vaccine induces a Th1 response, activates NK cells, and reduces renal colonization. Clin. Vaccine Immunol. 18, 684–691. 10.1128/CVI.00288-10 21288995 PMC3122574

