# Supplementary figures and images for: Inbred Rats as a Model to Study Persistent Renal Leptospirosis and Associated Cellular Immune Responsiveness
Source: Front Cell Infect Microbiol. 2018 Mar 14;8:66. doi: 10.3389/fcimb.2018.00066 (PMC5861151; doi:10.3389/fcimb.2018.00066)

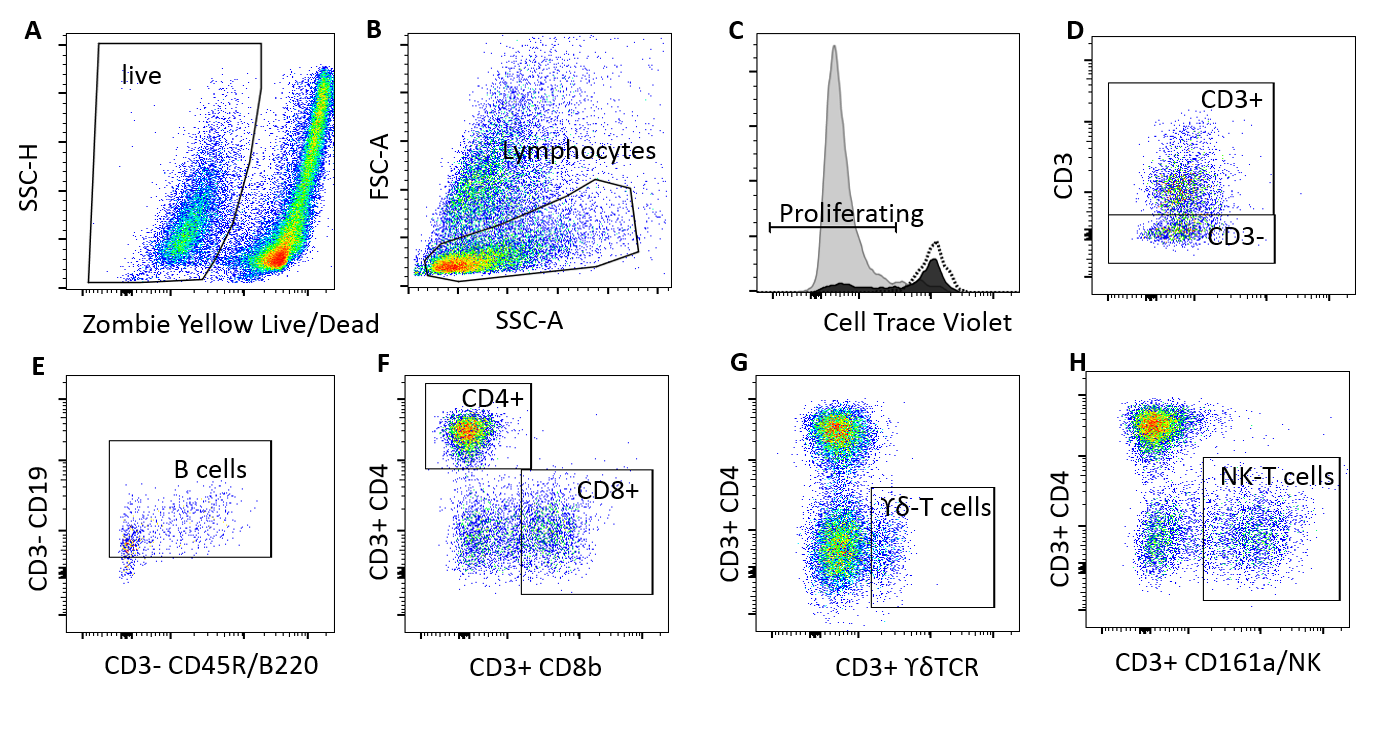

Supplement: Supplementary Figure 1 — Gating strategy for flow cytometry. A representative example using lymphocytes purified from the spleen of experimentally infected rats is provided. Live cells were gated using Zombie Yellow Live/Dead discriminator dye (A). Live cells were then gated on forward scatter (FSC) and side scatter (SSC) pattern consistent with proliferating lymphocytes (B). Proliferating lymphocytes were gated on decrease in fluorescence of Cell Trace Violet cellular proliferation dye (C). Dotted line is unstimulated cells, gray histogram is ConA 1 μg/ml stimulated cells and black is 0.5 μg/ml OM antigen stimulated cells. Proliferating cells were then gated into CD3+ and CD3– populations against an irrelevant marker (D). Proliferating B cell population was defined in CD3– subset as being CD19+ and/or CD45R/B220+ (E). Proliferating T cell subset (CD3+) was further phenotyped into CD4+ vs. CD8b+ (F), gamma-delta TCR+ (⋎δTCR) (G) and NK (CD161a+) (H) subsets respectively. [file Image1.TIF]
