# Supplementary material for: Inbred Rats as a Model to Study Persistent Renal Leptospirosis and Associated Cellular Immune Responsiveness
Source: Front Cell Infect Microbiol. 2018 Mar 14;8:66. doi: 10.3389/fcimb.2018.00066 (PMC5861151; doi:10.3389/fcimb.2018.00066)
Supplement: Supplementary Table 1 — Antibodies used for flow cytometry. [file Table1.DOCX]

Supplement Table 1 Number of significantly different pathways obtained with KEGG using the significantly changed genes in brains of Nile tilapia grown for 49 days in brackish water with a salinity of 16 psu versus those of tilapia in freshwater.

| Pathway | ID | Sample number | Background number | P-Value | Genes | Type II | Type I |  |  |
| --- | --- | --- | --- | --- | --- | --- | --- | --- | --- |
| Linoleic acid metabolism | ko00591 | 2 | 34 | 0.064 | 26630\|2608 | Lipid metabolism | Metabolism |  |  |
| Biotin metabolism | ko00780 | 1 | 5 | 0.067 | 10851 | Metabolism of cofactors and vitamins | Metabolism |  |  |
| Tropane, piperidine and pyridine alkaloid biosynthesis | ko00960 | 1 | 6 | 0.078 | 11903 | Biosynthesis of other secondary metabolites | Metabolism |  |  |
| Tyrosine metabolism | ko00350 | 2 | 40 | 0.084 | 14747\|11903 | Amino acid metabolism | Metabolism |  |  |
| Phototransduction | ko04744 | 2 | 45 | 0.101 | 12353\|4062 | Sensory system | Organismal Systems |  |  |
| Glycine, serine and threonine metabolism | ko00260 | 2 | 51 | 0.124 | 26795\|11903 | Amino acid metabolism | Metabolism |  |  |
| Cytosolic DNA-sensing pathway | ko04623 | 2 | 52 | 0.128 | 20071\|11230 | Immune system | Organismal Systems |  |  |
| Complement and coagulation cascades | ko04610 | 3 | 105 | 0.128 | 645\|2863\|2827 | Immune system | Organismal Systems |  |  |
| ECM-receptor interaction | ko04512 | 3 | 107 | 0.133 | 141\|1075\|2865 | Signaling molecules and interaction | Environmental Information Processing | | |
| Tryptophan metabolism | ko00380 | 2 | 54 | 0.135 | 487\|14747 | Amino acid metabolism | Metabolism |  |  |
| Lysosome | ko04142 | 4 | 168 | 0.137 | 3460\|2754\|7402\|2770 | Transport and catabolism | Cellular Processes |  |  |
| Ubiquinone and other terpenoid-quinone biosynthesis | ko00130 | 1 | 12 | 0.139 | 20085 | Metabolism of cofactors and vitamins | Metabolism |  |  |
| Phenylalanine, tyrosine and tryptophan biosynthesis | ko00400 | 1 | 12 | 0.139 | 14747 | Amino acid metabolism | Metabolism |  |  |
| Metabolism of xenobiotics by cytochrome P450 | ko00980 | 2 | 56 | 0.143 | 10021\|15663 | Xenobiotics biodegradation and metabolism | Metabolism |  |  |
| Prolactin signaling pathway | ko04917 | 3 | 113 | 0.149 | 1493\|6469\|6467 | Endocrine system | Organismal Systems |  |  |
| Streptomycin biosynthesis | ko00521 | 1 | 13 | 0.149 | 6350 | Biosynthesis of other secondary metabolites | Metabolism |  |  |
| Protein digestion and absorption | ko04974 | 4 | 175 | 0.151 | 19207\|141\|1075\|18739 | Digestive system | Organismal Systems |  |  |
| Alanine, aspartate and glutamate metabolism | ko00250 | 2 | 59 | 0.155 | 13006\|14747 | Amino acid metabolism | Metabolism |  |  |
| Toll-like receptor signaling pathway | ko04620 | 3 | 116 | 0.157 | 14114\|20071\|11230 | Immune system | Organismal Systems |  |  |
| Cysteine and methionine metabolism | ko00270 | 2 | 61 | 0.163 | 14747\|26795 | Amino acid metabolism | Metabolism |  |  |
| Retinol metabolism | ko00830 | 2 | 62 | 0.167 | 15663\|9582 | Metabolism of cofactors and vitamins | Metabolism |  |  |
| ABC transporters | ko02010 | 2 | 63 | 0.171 | 17076\|20042 | Membrane transport | Environmental Information Processing | | |
| Natural killer cell mediated cytotoxicity | ko04650 | 4 | 185 | 0.173 | 3482\|18447\|1466\|9313 | Immune system | Organismal Systems |  |  |
| Bile secretion | ko04976 | 3 | 125 | 0.182 | 10021\|14364\|9444 | Digestive system | Organismal Systems |  |  |
| Vasopressin-regulated water reabsorption | ko04962 | 2 | 67 | 0.188 | 15957\|15235 | Excretory system | Organismal Systems |  |  |
| Fatty acid biosynthesis | ko00061 | 1 | 20 | 0.215 | 16924 | Lipid metabolism | Metabolism |  |  |
| GnRH signaling pathway | ko04912 | 3 | 137 | 0.217 | 20373\|1493\|5826 | Endocrine system | Organismal Systems |  |  |
| Steroid biosynthesis | ko00100 | 1 | 21 | 0.224 | 6891 | Lipid metabolism | Metabolism |  |  |
| Terpenoid backbone biosynthesis | ko00900 | 1 | 22 | 0.233 | 8564 | Metabolism of terpenoids and polyketides | Metabolism |  |  |
| Arachidonic acid metabolism | ko00590 | 2 | 79 | 0.238 | 26630\|2608 | Lipid metabolism | Metabolism |  |  |
| PPAR signaling pathway | ko03320 | 2 | 79 | 0.238 | 20885\|16924 | Endocrine system | Organismal Systems |  |  |
| Intestinal immune network for IgA production | ko04672 | 2 | 85 | 0.264 | 19932\|25111 | Immune system | Organismal Systems |  |  |
| Inflammatory mediator regulation of TRP channels | ko04750 | 3 | 153 | 0.266 | 26630\|2608\|11230 | Sensory system | Organismal Systems |  |  |
| Ascorbate and aldarate metabolism | ko00053 | 1 | 28 | 0.284 | 15663 | Carbohydrate metabolism | Metabolism |  |  |
| Synaptic vesicle cycle | ko04721 | 2 | 91 | 0.289 | 20057\|10966 | Nervous system | Organismal Systems |  |  |
| Serotonergic synapse | ko04726 | 3 | 162 | 0.294 | 26630\|2608\|2145 | Nervous system | Organismal Systems |  |  |
| beta-Alanine metabolism | ko00410 | 1 | 34 | 0.332 | 11903 | Metabolism of other amino acids | Metabolism |  |  |
| Adipocytokine signaling pathway | ko04920 | 2 | 104 | 0.344 | 18737\|16924 | Endocrine system | Organismal Systems |  |  |
| Vitamin digestion and absorption | ko04977 | 1 | 38 | 0.363 | 10851 | Digestive system | Organismal Systems |  |  |
| Biosynthesis of unsaturated fatty acids | ko01040 | 1 | 38 | 0.363 | 15077 | Lipid metabolism | Metabolism |  |  |
| Porphyrin and chlorophyll metabolism | ko00860 | 1 | 39 | 0.37 | 15663 | Metabolism of cofactors and vitamins | Metabolism |  |  |
| Pentose and glucuronate interconversions | ko00040 | 1 | 39 | 0.37 | 15663 | Carbohydrate metabolism | Metabolism |  |  |
| Pyrimidine metabolism | ko00240 | 2 | 113 | 0.381 | 7165\|3657 | Nucleotide metabolism | Metabolism |  |  |
| SNARE interactions in vesicular transport | ko04130 | 1 | 42 | 0.391 | 10966 | Folding, sorting and degradation | Genetic Information Processing | |  |
| Fatty acid elongation | ko00062 | 1 | 44 | 0.405 | 15077 | Lipid metabolism | Metabolism |  |  |
| Proteasome | ko03050 | 1 | 48 | 0.432 | 20043 | Folding, sorting and degradation | Genetic Information Processing | |  |
| Proximal tubule bicarbonate reclamation | ko04964 | 1 | 49 | 0.439 | 9444 | Excretory system | Organismal Systems |  |  |
| Drug metabolism - cytochrome P450 | ko00982 | 1 | 50 | 0.445 | 15663 | Xenobiotics biodegradation and metabolism | Metabolism |  |  |
| Fatty acid degradation | ko00071 | 1 | 51 | 0.451 | 16924 | Lipid metabolism | Metabolism |  |  |
| Phosphatidylinositol signaling system | ko04070 | 2 | 133 | 0.46 | 19254\|6350 | Signal transduction | Environmental Information Processing | | |
| Fanconi anemia pathway | ko03460 | 1 | 55 | 0.476 | 181 | Replication and repair | Genetic Information Processing | |  |
| Drug metabolism - other enzymes | ko00983 | 1 | 56 | 0.482 | 15663 | Xenobiotics biodegradation and metabolism | Metabolism |  |  |
| Steroid hormone biosynthesis | ko00140 | 1 | 56 | 0.482 | 15663 | Lipid metabolism | Metabolism |  |  |
| Valine, leucine and isoleucine degradation | ko00280 | 1 | 58 | 0.494 | 14747 | Amino acid metabolism | Metabolism |  |  |
| Starch and sucrose metabolism | ko00500 | 1 | 59 | 0.5 | 15663 | Carbohydrate metabolism | Metabolism |  |  |
| Phospholipase D signaling pathway | ko04072 | 3 | 233 | 0.51 | 19254\|7953\|15235 | Signal transduction | Environmental Information Processing | | |
| Fatty acid metabolism | ko01212 | 1 | 62 | 0.517 | 16924 | Global and overview maps | Metabolism |  |  |
| Adherens junction | ko04520 | 2 | 154 | 0.536 | 15142\|13988 | Cellular community - eukaryotes | Cellular Processes |  |  |
| Regulation of actin cytoskeleton | ko04810 | 4 | 332 | 0.539 | 4788\|10381\|02145\|25963 | Cell motility | Cellular Processes |  |  |
| Amino sugar and nucleotide sugar metabolism | ko00520 | 1 | 67 | 0.544 | 13006 | Carbohydrate metabolism | Metabolism |  |  |
| Ubiquitin mediated proteolysis | ko04120 | 2 | 163 | 0.566 | 5018\|11045 | Folding, sorting and degradation | Genetic Information Processing | |  |
| Endocytosis | ko04144 | 5 | 439 | 0.578 | 3482\|18447\|26254\|1466\|6024 | Transport and catabolism | Cellular Processes |  |  |
| Hematopoietic cell lineage | ko04640 | 1 | 76 | 0.589 | 11230 | Immune system | Organismal Systems |  |  |
| Glycerolipid metabolism | ko00561 | 1 | 76 | 0.589 | 19254 | Lipid metabolism | Metabolism |  |  |
| RIG-I-like receptor signaling pathway | ko04622 | 1 | 77 | 0.594 | 20071 | Immune system | Organismal Systems |  |  |
| Ribosome biogenesis in eukaryotes | ko03008 | 1 | 78 | 0.599 | 20798 | Translation | Genetic Information Processing | |  |
| Sphingolipid metabolism | ko00600 | 1 | 82 | 0.617 | 24161 | Lipid metabolism | Metabolism |  |  |
| NOD-like receptor signaling pathway | ko04621 | 1 | 82 | 0.617 | 11230 | Immune system | Organismal Systems |  |  |
| RNA degradation | ko03018 | 1 | 85 | 0.63 | 370 | Folding, sorting and degradation | Genetic Information Processing | |  |
| Dopaminergic synapse | ko04728 | 2 | 196 | 0.665 | 2145\|14071 | Nervous system | Organismal Systems |  |  |
| Renin secretion | ko04924 | 1 | 98 | 0.681 | 9444 | Endocrine system | Organismal Systems |  |  |
| Biosynthesis of amino acids | ko01230 | 1 | 100 | 0.689 | 26795 | Global and overview maps | Metabolism |  |  |
| Regulation of lipolysis in adipocytes | ko04923 | 1 | 100 | 0.689 | 24421 | Endocrine system | Organismal Systems |  |  |
| Focal adhesion | ko04510 | 3 | 307 | 0.692 | 141\|1075\|2865 | Cellular community - eukaryotes | Cellular Processes |  |  |
| Inositol phosphate metabolism | ko00562 | 1 | 105 | 0.706 | 6350 | Carbohydrate metabolism | Metabolism |  |  |
| Thyroid hormone synthesis | ko04918 | 1 | 105 | 0.706 | 24421 | Endocrine system | Organismal Systems |  |  |
| Purine metabolism | ko00230 | 2 | 214 | 0.711 | 8339\|7165 | Nucleotide metabolism | Metabolism |  |  |
| Olfactory transduction | ko04740 | 1 | 113 | 0.732 | 4120 | Sensory system | Organismal Systems |  |  |
| NF-kappa B signaling pathway | ko04064 | 1 | 114 | 0.735 | 11230 | Signal transduction | Environmental Information Processing | | |
| Cardiac muscle contraction | ko04260 | 1 | 118 | 0.747 | 20696 | Circulatory system | Organismal Systems |  |  |
| Glycerophospholipid metabolism | ko00564 | 1 | 120 | 0.753 | 19254 | Lipid metabolism | Metabolism |  |  |
| Apoptosis | ko04210 | 1 | 123 | 0.762 | 11230 | Cell growth and death | Cellular Processes |  |  |
| GABAergic synapse | ko04727 | 1 | 126 | 0.77 | 2145 | Nervous system | Organismal Systems |  |  |
| Gap junction | ko04540 | 1 | 135 | 0.792 | 12747 | Cellular community - eukaryotes | Cellular Processes |  |  |
| Insulin secretion | ko04911 | 1 | 136 | 0.795 | 13246 | Endocrine system | Organismal Systems |  |  |
| MAPK signaling pathway | ko04010 | 3 | 369 | 0.802 | 9756\|2145\|11230 | Signal transduction | Environmental Information Processing | | |
| TNF signaling pathway | ko04668 | 1 | 142 | 0.809 | 11230 | Signal transduction | Environmental Information Processing | | |
| Spliceosome | ko03040 | 1 | 145 | 0.815 | 370 | Transcription | Genetic Information Processing | |  |
| Melanogenesis | ko04916 | 1 | 145 | 0.815 | 18737 | Endocrine system | Organismal Systems |  |  |
| Fc gamma R-mediated phagocytosis | ko04666 | 1 | 149 | 0.824 | 10381 | Immune system | Organismal Systems |  |  |
| Retrograde endocannabinoid signaling | ko04723 | 1 | 157 | 0.839 | 2145 | Nervous system | Organismal Systems |  |  |
| Circadian entrainment | ko04713 | 1 | 161 | 0.846 | 2145 | Environmental adaptation | Organismal Systems |  |  |
| Osteoclast differentiation | ko04380 | 1 | 162 | 0.848 | 11230 | Development | Organismal Systems |  |  |
| Pancreatic secretion | ko04972 | 1 | 166 | 0.855 | 3453 | Digestive system | Organismal Systems |  |  |
| RNA transport | ko03013 | 1 | 168 | 0.858 | 20798 | Translation | Genetic Information Processing | |  |
| Neurotrophin signaling pathway | ko04722 | 1 | 175 | 0.869 | 9756 | Nervous system | Organismal Systems |  |  |
| Vascular smooth muscle contraction | ko04270 | 1 | 176 | 0.871 | 15235 | Circulatory system | Organismal Systems |  |  |
| Insulin signaling pathway | ko04910 | 1 | 181 | 0.878 | 13988 | Endocrine system | Organismal Systems |  |  |
| Cholinergic synapse | ko04725 | 1 | 186 | 0.885 | 2145 | Nervous system | Organismal Systems |  |  |
| Glutamatergic synapse | ko04724 | 1 | 188 | 0.888 | 2145 | Nervous system | Organismal Systems |  |  |
| cAMP signaling pathway | ko04024 | 2 | 336 | 0.901 | 5826\|13246 | Signal transduction | Environmental Information Processing | | |
| FoxO signaling pathway | ko04068 | 1 | 200 | 0.902 | 15450 | Signal transduction | Environmental Information Processing | | |
| Signaling pathways regulating pluripotency of stem cells | ko04550 | 1 | 204 | 0.907 | 2652 | Cellular community - eukaryotes | Cellular Processes |  |  |
| Chemokine signaling pathway | ko04062 | 1 | 246 | 0.943 | 2145 | Immune system | Organismal Systems |  |  |
| Adrenergic signaling in cardiomyocytes | ko04261 | 1 | 247 | 0.943 | 20696 | Circulatory system | Organismal Systems |  |  |
| Calcium signaling pathway | ko04020 | 1 | 295 | 0.968 | 16886 | Signal transduction | Environmental Information Processing | | |
| Ras signaling pathway | ko04014 | 1 | 309 | 0.972 | 2145 | Signal transduction | Environmental Information Processing | | |
